# Characterization and flowability methods for metal powders

**DOI:** 10.1038/s41598-020-77974-3

**Published:** 2020-12-03

**Authors:** Jiri Zegzulka, Daniel Gelnar, Lucie Jezerska, Rostislav Prokes, Jiri Rozbroj

**Affiliations:** 1 ENET Centre, Bulk Solids Centre, VSB-TU Ostrava, 17. listopadu 15, 70800 Ostrava, Czech Republic; 2 Department of Mining Engineering and Safety, Faculty of Mining and Geology, VSB-TU Ostrava, 17. listopadu 15, 70800 Ostrava, Czech Republic

**Keywords:** Engineering, Materials science, Mathematics and computing, Particle physics

## Abstract

With the rise of additive technologies, the characterization of metal powders is increasingly required. There is a need to precisely match the properties of metal powders to a specific machine and to ensure highly consistent production. Therefore, the study aims at a detailed characterization of ten metal powders (Metal powder 316 L, Zn, Sn, Al, Cu, Mn, Fe, Bronze, Ti and Mo powder), for which the particle size distribution, morphology, static and dynamic angle of repose and the effective internal friction angle (AIFE) were determined. The AIFE parameter and flow index were determined from three commonly used rotary shear devices: The computer-controlled Ring Shear Tester RST-01. pc, the Brookfield PFT Powder Flow Tester and the FT4 Powder rheometer. The results showed that the values ​​for the device of one manufacturer did not fully correspond to the values ​​of another one. The flow characteristics of the metal powders were quantified from the particle size distribution data, static angle of repose, and AIFE data. According to the particle size distribution and angle of repose (AOR), 50% of the tested metal powders fell into the free-flowing mode. According to the evaluation of AIFE, 20% of the samples fell into the lower area. Based on the flow indexes calculated from the measurements of the shear devices used, 100% (RST-01.pc), 70% (PFT) and 50% (FT4) of the samples were included in the free-flowing category. When comparing the results, attention should be paid not only to the nature of the material, but also to the methodology and equipment used. A comparison of methodologies revealed similarities in the changing behavior of the different metal powders. A comparison of effective angles of AIFE and static AOR was shown, and a hypothesis of the conversion relation was derived.

## Introduction

Metal parts can be obtained using a variety of traditional and modern techniques. Most of these modern techniques use metal powders as a starting material, which are further processed into various end products. Metal powders can be processed, for example, by pressing, sintering, thermal spraying techniques or more modern additive manufacturing techniques, such as selective laser melting, SLM^1^, laser powder-fed LPF^2^, binder jetting BJ^3,4^, electron-beam powder-bed fusion EPBF^5^ and others^6,7^. The choice of the technological production process leads, of course, to different qualitative properties of the final products, but for all techniques the input characterization of the metal powders used is important. Among the parameters determined for powders are flowability, chemical composition, particle size, optical properties, thermo-physical properties, surface tension and others^7^. Every additive production process has its own requirements for specific parameters of metal powders.

The characteristics of powders having a significant effect on the final metal product, but also on the technological process, are, for example, particle shape, particle size, flowability and the ratio of the mass of the powder to the volume occupied by the powder after it has been tapped for a defined period, called as tap density^7,8^. The tap density of a powder represents its random dense packing. For example, a sufficient tap density will help to ensure sufficient packing of powder layers and high green density^9^. The opposite can be considerable porosity and subsequent high shrinkage during sintering. This can lead to the problem of achieving the desired density of the final product^10^.

A spherical shape of particles is welcome in the field of AM technologies. Sphericity is a significant advantage for good flowability of metal powders, even if irregular particles are able to improve green strength without achieving uniform density^10^.

Particle size distribution is another important parameter. For example, a wide particle size distribution can affect packing behaviors and consequently also shrinkage and densification of moulding parts^11^. In another study, it was shown that the particle size distribution affects the sintering behavior of complexly shaped biomedical parts 316L SS^12^.

**Figure 1:**
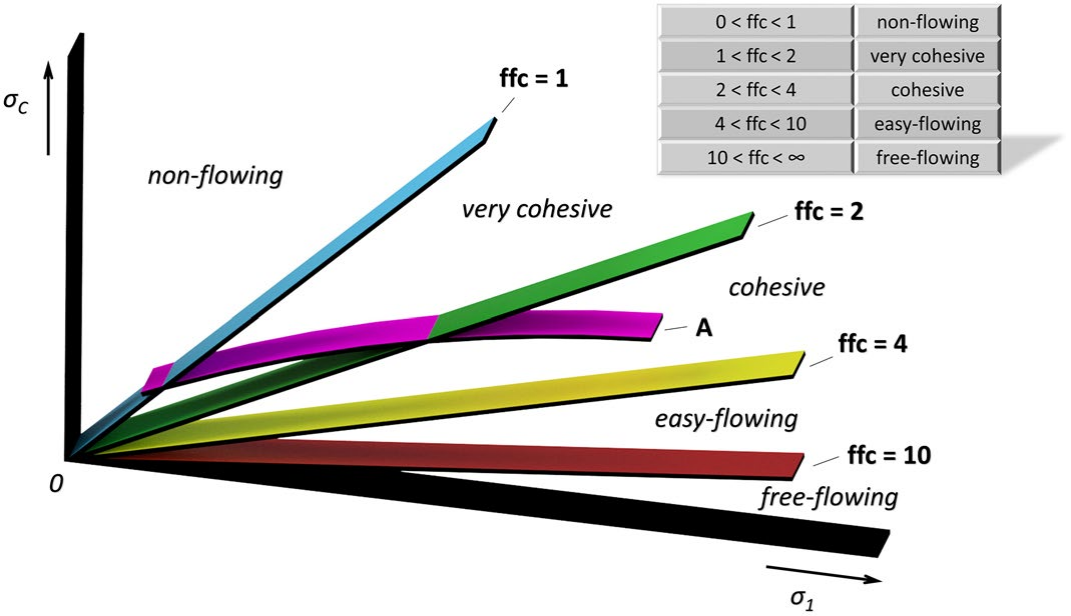
Range of different flowability levels, flowability classification.

The flowability of metal powders is not an inherent property – it depends not only on the physical properties (shape, particle size, humidity, etc.), but also on the stress state, the equipment used and the handling method^13,14^. The flow of powders in individual methods of additive technologies is a complex area of study. Powder companies would like to avoid flow problems such as segregation, vaulting, agglomeration, and would like to predict how a particular metal powder will flow and form/not form a homogeneous layer, or compare the flow characteristics of metal powders with each other. Due to the price of metal powders, only a limited amount is provided for testing, so it is beneficial to test new types of metal powders in the laboratory. However, a uniform and comprehensive way of describing the flow of metal powders does not yet exist^15^. Therefore, it is necessary to consider the possibilities of experimental determination techniques, to compare the quality of equipment and measured results. Several experimental techniques have been developed to determine the flow rate of powders in general—calculation of the Carrs index and Hausner ratio from tap and bulk density values^16^, using a static or dynamic AOR^16,17^ or the most commonly used shear cell measurement technique and subsequent classification of powders according to Jenike^19^. Static and dynamic AOR is significantly affected by the particle shape and size^20,21^, moisture content^22^, bulk density^21,23,24^ and the action of gravity^25,26^. For flowability classification, Jenike proposed the flow index *ffc*, which is the ratio of the major principal stress σ_1_ at steady state flow to the unconfined yield strength σ_c_. This classification was expanded by Tomas, as shown in Fig. 1^27^.

Curve A presents the frequently-occurring case of a powder where a slow growth of the unconfined yield strength σ_c_ increases with the growing major consolidation stress σ_1_. Depending on load, a material falls into one of the following categories: non-flowing, very cohesive, or cohesive. Shear cells are now commonly used to rank powder materials according to their flowability^28^. The shear cell can also be used to measure the bulk density of a powders as a function of applied normal stress. Bulk density includes both particle density and information on the packing of the powder bed^29^. The AIFE can be determined from the shear tests. As flow characterization became more widespread, standards describing the testing procedure were also defined^30,31^. Although there are many studies on shear cell measurements^32–35^, only a limited number of publications are devoted to comparing measurements between different types of shear testers^36–39^. For example, Schulze^36^ reports a study where similar results were obtained for shear cells of different sizes—30 and 900 ml. Koynov^38^ compared the flowability and bulk density of free-flowing and cohesive powder using three rotary shear testers. These were the PFT, RST and FT4. From the paper’s conclusion, it followed that it is the type of material tested that has the most significant effect on shear cell results. In addition, the consolidation at which the material was tested and the tester type have statistically significant effects^38^. Salehi^39^ compared the Jenike, Schulze and Brookfield PFT shear testers at the same load conditions. There were used three flowability different powders: moderate cohesive dolomitic lime, free-flowing calcium lactate and very cohesive calcium carbonate. The study showed that the good agreement between testers in terms of shear stresses is for powder from moderately cohesive and free-flowing areas. The largest differences between the testers were found for very cohesive calcium carbonate. The degree of agreement between shear testers is highly dependent on the used powders and does not apply across the flowability classes. Other results from Jenike tester indicated, that some care should be given to the interpretation of the pre-shear data. However, many questions still remain as to whether the measurement of flowability, or the AIFE, respectively, in one device in a given shear cell will correspond to the same measurement in a device from another manufacturer. Given the growing need for characterization of both existing and new metal powders in terms of flow in the field of additive technologies, it is important to compare the results of several instruments and thus answer the questions of measurement accuracy and comparability.

Therefore, the article aims to address two cases: the characterization of 10 metal powders in terms of particle size distribution, particle shape, static AOR, dynamic AOR and the comparison of three devices for measuring the AIFE, or flow rates of metal powders. First, three commonly used devices are briefly described: The computer-controlled Ring Shear Tester RST-01.pc (RST), Brookfield Powder Flow Tester (PFT) and the FT4 Powder rheometer (FT4). A characterization of 10 metal powders follows. The resulting data obtained using these methods is then summarized in the second part of the article. Overall comparisons and recommendations are presented in the conclusion of the paper’s conclusion.

## Materials and methods

### Materials

Ten different metal powders were used for experiments—metal powder 316L, zinc (Zn), tin (Sn), aluminum (Al), copper (Cu), manganese (Mn), iron (Fe), Bronze, titanium (Ti) and molybdenum (Mo) powder.

The sample of metal powder 316L was taken from a 3D printing manufacturing operation, tin and molybdenum powders are commercially available from Svět Prvků s.r.o., manganese powder was purchased through pkchemie-kovyachemie.cz and other metal powders were purchased from Fichema s.r.o.

### Methods

#### Particle size distribution and particle shape

Granulometric analysis of the metallic powder sample was performed on the Cilas 1190 (Anton Paar, Les Ulis, France) laser analyser. Laser diffraction for particle size measurement is widely used for many different types of particles across many different industries^17^. The wet path method was used. The medium used in the tests was water. The metal powders were measured after sonication to ensure complete dispersion. Determination of the particles proceeded on the basis of the passage of the measured material dispersed in the carrier medium through coherent light with a wavelength of 830 nm. The results were interpreted based on the Fraunhofer theory^18^.

One measurement was repeated 10 times. The resulting parameters d_10_, d_50_ and d_90_ are the average values. Span S (Eq. 1) represents the width of the Gauss distribution layout based on the metric calculation^19^.

1 $$S = \frac{{d_{90} - d_{10} }}{{d_{50} }},$$

The particles shape was evaluated by a scanning electron microscope (SEM, FEI QUANTA 650 FEG). SEM is a non-destructive imaging technique to study the micro structural of the particles. To analyse the micro structure of the particles by using the high-energy electrons focused beam to produce a different signals at the particle surface. The photographs were taken under pressure of 3·10^−3^ Pa and HV of 20.00 kV.

### Flowability of metal powders

The flow rate of metal powders was determined using a static AOR (Table 1), which was supplemented by a changing the AOR to describe the changing behavior of the samples. Furthermore, the flowability of metal powders was determined based on shear tests—using RST, PFT and FT4 devices.

**Table 1:** Classification of flow properties by the AOR.

AOR	Flow properties
20° < α < 30°	Very free-flowing
30° < α < 38°	Free flowing
38° < α < 45°	Fair to passable flow
45° < α < 55°	Cohesive
55° < α < 70°	Very cohesive

#### Static AOR (Zenegero)

**Figure 2:**
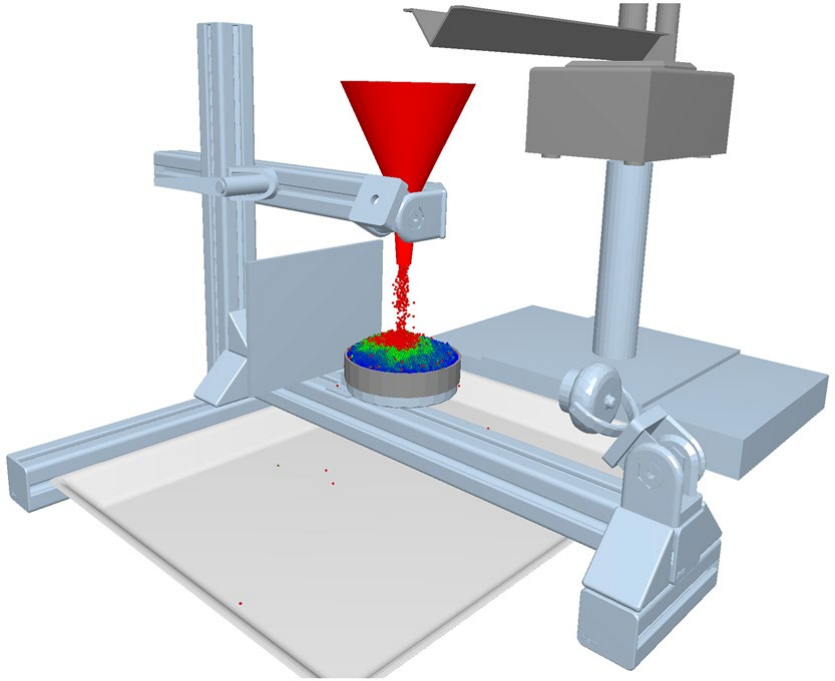
Device for setting AOR.

A patented device was used to determine the static AOR of metal powders, based on the patent PV2015-239. The validation of the device and method of measuring static and dynamic angle of discharge, VSB – Technical University of Ostrava, is shown on Fig. 2, the so-called Zenegero^40^.

At the beginning, the tested metal powder is gradually fed to a stainless-steel rotating dish by a vibrating feeder and a conical hopper. The material is continuously weighed so that it is possible to determine the moment when there is no further weight increase (additional material is poured out of the stainless-steel rotating bowl down the slope onto the base). The camera then captures an image of the slope from 8 different sides. The average slope angle was evaluated by graphical post processing.

Classification of powder flow according to AOR values is evident in Table 1^41,42^. Powders can be categorized in to the 5 following groups. In general, it applies that a smaller AOR indicates better flow properties as found in the Table 1.

#### Dynamic AOR

A dynamic AOR, in our case representing the changing behavior of metal powders, was measured in a rotating vessel with a diameter of 0.140 m and a width of 0.03 m^43^. The fill level was 45%. The rotation frequency was set to 0.2, 0.4 and 0.6 Hz. The dynamic AOR was evaluated 10 times for all materials for each set frequency. The stability characteristics of the angles for individual frequencies, but also in general out of all frequencies, were assessed for individual samples. Three different characteristics of flow stability were determined, and the individual samples were also assessed amongst each other. Based on the dynamic behavior of metal powders during the tests, the types of powder movements in a rotating vessel were described and classified.

#### Span of particle size distribution

Powders with larger size distribution span (Eq. 1, “Particle size distribution and particle shape” section) induced reductions in flowability^44^. The limit value is 1.5. The powders with value of Span S ≤ 1.5 exhibit good flow (group 1), when S ≫  > 1.5 the powder displayed resistance to flow (group 2).

#### Shear cell procedures—AIFE and flow index

- RST measurement

**Figure 3:**
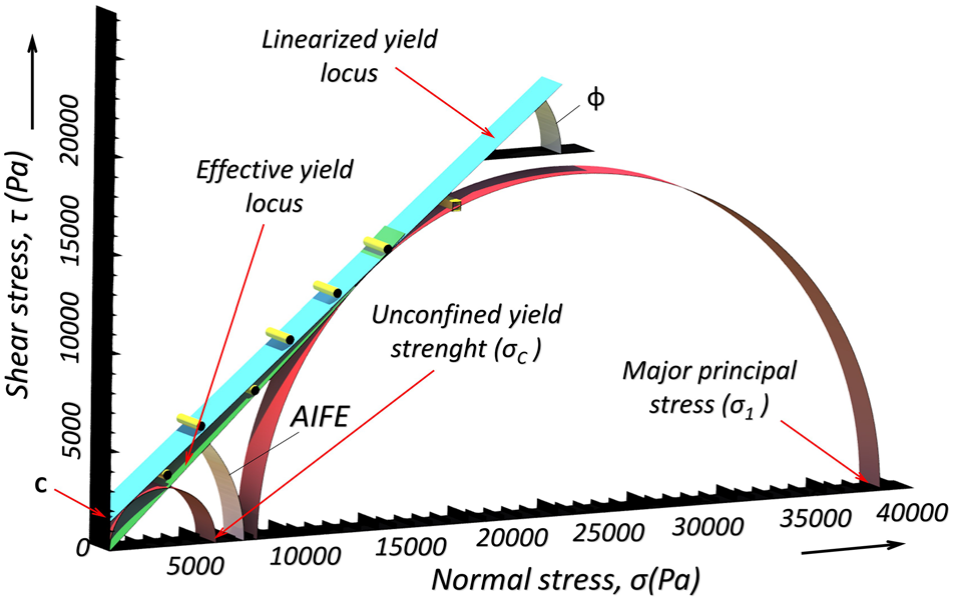
Schematic diagram of powder yield locus obtained from the Schulze ring tester^45^.

The first measurement of the AIFE resp. flowability evaluation was performed on the RST-01.pc (Dietmar Schulze, Wolggenbuttel, Germany). The principle of measurement of the angle of AIFE consists in measuring the time dependence of the shear force which is required for the transformation of the bulk solid in a shear chamber through the shear zone under the influence of normal load, for a specific density of the bulk material^34^. The density for the given measurement is achieved through consolidation (compaction) at a defined strength load. Shear force is applied by the rotating chamber of the apparatus, and the torque is transmitted by two rods which are fixed onto the shear lid during the rotational measurement test. Figure 3^45^ shows a powder yield locus obtained from measurements on a RST, which contains a great deal of important information, including the linearized angle of internal friction *ϕ*, cohesion *c*, effective angle of internal friction (AIFE), major consolidation stress σ_1_ and the unconfined yield strength σ_c_.

The AIFE was measured for each metal powder 10 times for 10,000 Pa normal stress settings. The resulting AIFE for individual metal powders are the average values ​​of 10 measurements. The software related to shear cell was used to derive flow properties.

The relationship between the unconfined yield strength σ_c_ and the major principal stress σ_1_ is called the flow index *ffc* of the powders as mentioned in introduction section. The metal powders were characterized by the ratio *ffc* = σ_1_/ σ_c_. The larger *ffc*, the more easily the bulk materials flow^27,46,47^.

- PFT measurement (PFT)

**Figure 4:**
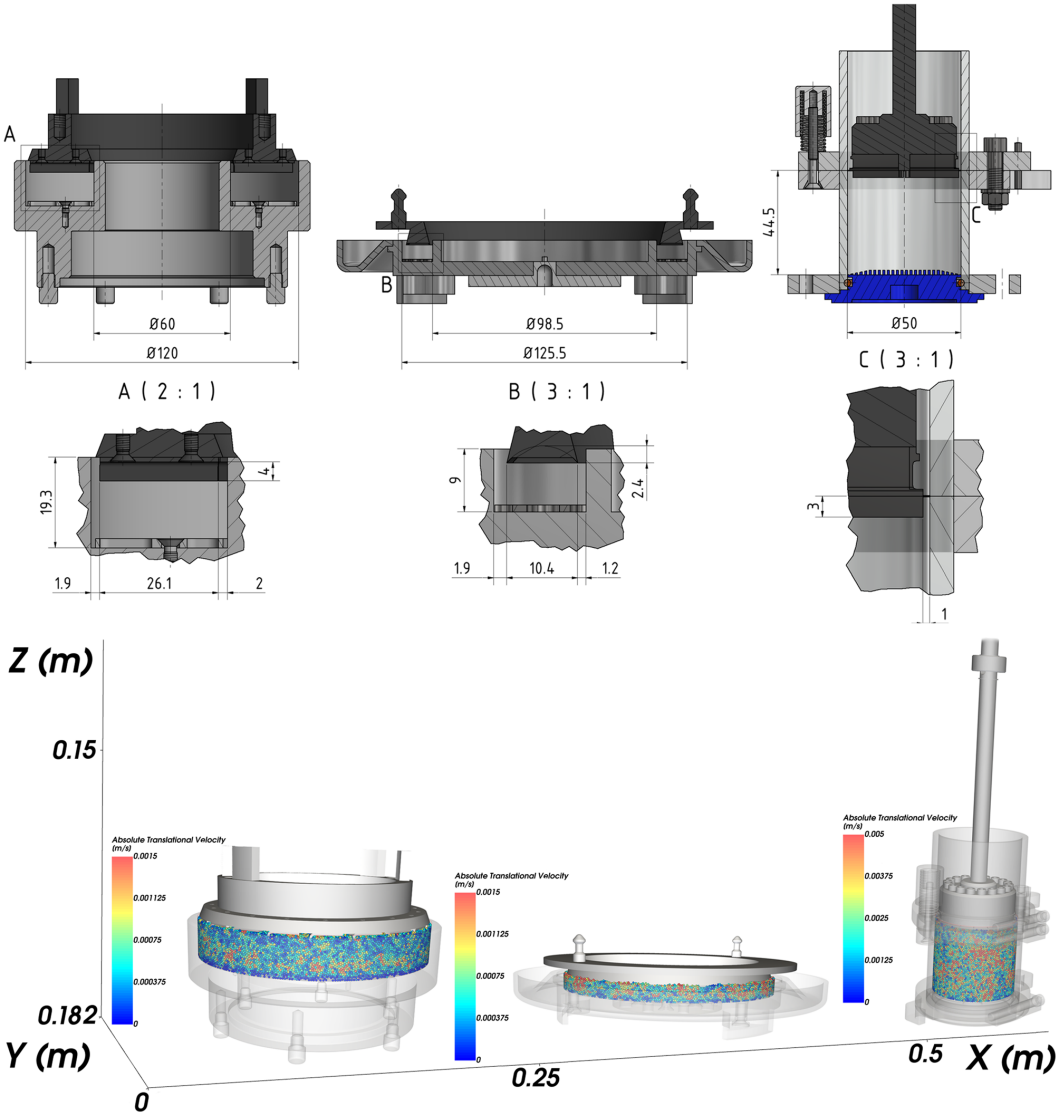
The shear cells. Left—RST, middle—PFT, right—FT4.

The second measurement of the AIFE resp. flowability evaluation was performed on the Powder Flow Tester (Brookfield Engineering Laboratories, Ing., Middleboro, MA, USA)^48,49^. The principle of PFT measurement is based on the movement of the compression lid vertically downwards towards the loose sample located in the circular shear cell. The volume of the bulk sample is defined by the shear cell used; in our case a small cell was used (Fig. 4). The weight of the sample is determined before starting the test. A calibrated beam load cell is used to precisely control the compressive stress acting on the bulk material. The measurement takes place during the rotation of the bulk material with the shear cell at the defined speed. The reading of the torsional resistances of the bulk material are taken in a stationary lid using a calibrated torsion sensor. For calculations that define the flow property of the bulk material, the dimensions of the shear cell lid (Fig. 4), the cell rotation speed (1 mm s^−1^) and the normal load acting on the bulk material are taken into account. To measure the AIFE, a predefined standard flow index test was used. The maximum normal load was chosen to be 10,000 Pa.

- FT4 measurement

The third measurement of the AIFE resp. flowability evaluation was performed on the FT4 (Freeman Technology, Tewkesbury, Gloucestershire, UK). The rotary shear module for measuring friction parameters consists of a vessel containing the sample powder and a shear head to cause normal and shear stress^50^. The blades of the shear head sink into the mass powder and the front face of the head starts to apply normal stress to the surface of the powder bed. The shear head moves downwards until a sufficient and stable pressure is applied between the head and powder bed. Then the shear head starts to rotate slowly and thus cause shear stress within the bulk mass. The shear plane is formed just below the end of the blades. Since the powder bed prevents the rotation of the shear head, the shear stress in the measuring plane increases until slippage occurs. Then, the maximum value of transferred shear stress is recorded. The AIFE was again measured for standard (consolidated) stress—10,000 Pa. The software relate to device was used to derive the flow index *ffc*.

- Technical comparison of shear cells used

The shear cells used in the study are shown in detail on Fig. 4. All three shear cells are rotational. However, there are differences in the measurement processes and characteristics of the shear cells, such as geometry, cell size, area ratios (Fig. 4), and particle quantity and overall volume of the measured sample.

RST has the largest shear cell area (8482 mm^2^), followed by PFT (4750 mm^2^) and finally FT4 (1879 mm^2^). The level of the bed in the shear cell was highest for FT4 (44.5 mm), then RST (19.3 mm), and finally for PFT (9 mm). The effect of difference in construction design of the shear cells for shear strength σ_c_, and therefore also on *ffc* values, was examined.

## Results and discussion

### Particle size distributions and particle shape

**Figure 5:**
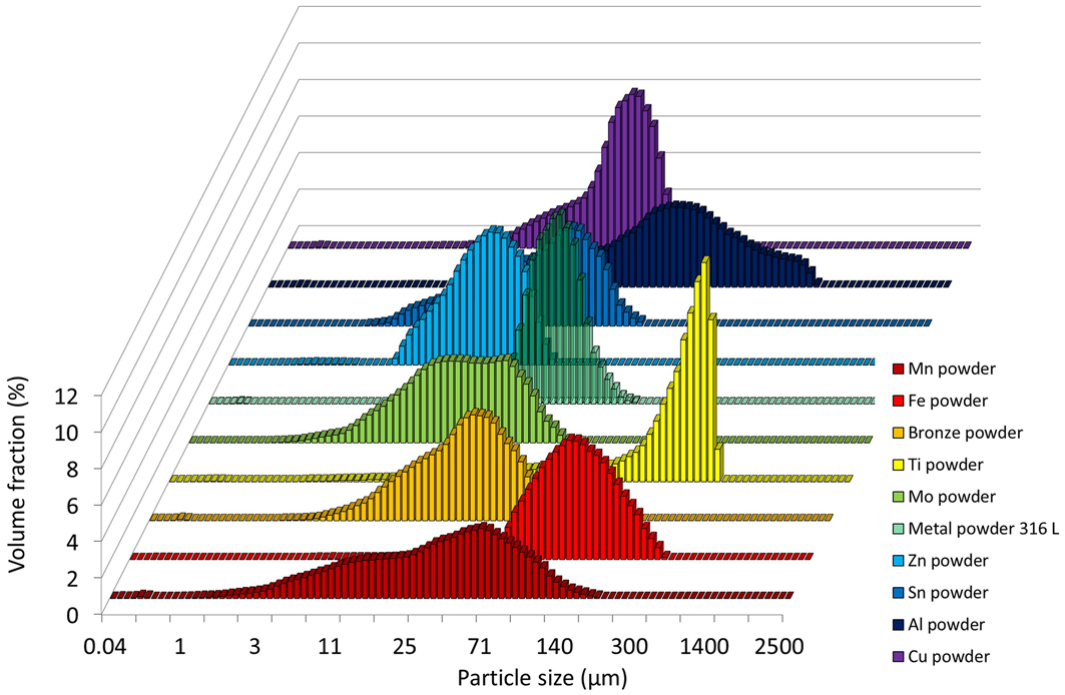
The particle size distribution for the then metal powders.

The particle size distributions of the first five metal powders are shown in Fig. 5. The values are shown in Table 2.

**Table 2:** Characteristics values for the particle size distribution of metal powders.

	d10, µm	d50, µm	d90, µm	Span S, –
Metal powder 316 L	26.7	42.6	64.0	0.9
Zn powder	8.3	15.8	24.8	1.0
Sn powder	8.5	25.9	53.6	1.7
Al powder	29.4	79.1	187.3	2.0
Cu powder	16.1	35.6	57.5	1.2
Mn powder	6.1	32.3	88.0	2.5
Fe powder	72.7	121.1	195.1	1.0
Bronze powder	12.1	29.6	57.2	1.5
Ti powder	80.7	295.1	452.1	1.3
Mo powder	6.0	18.1	44.9	2.1

**Figure 6:**
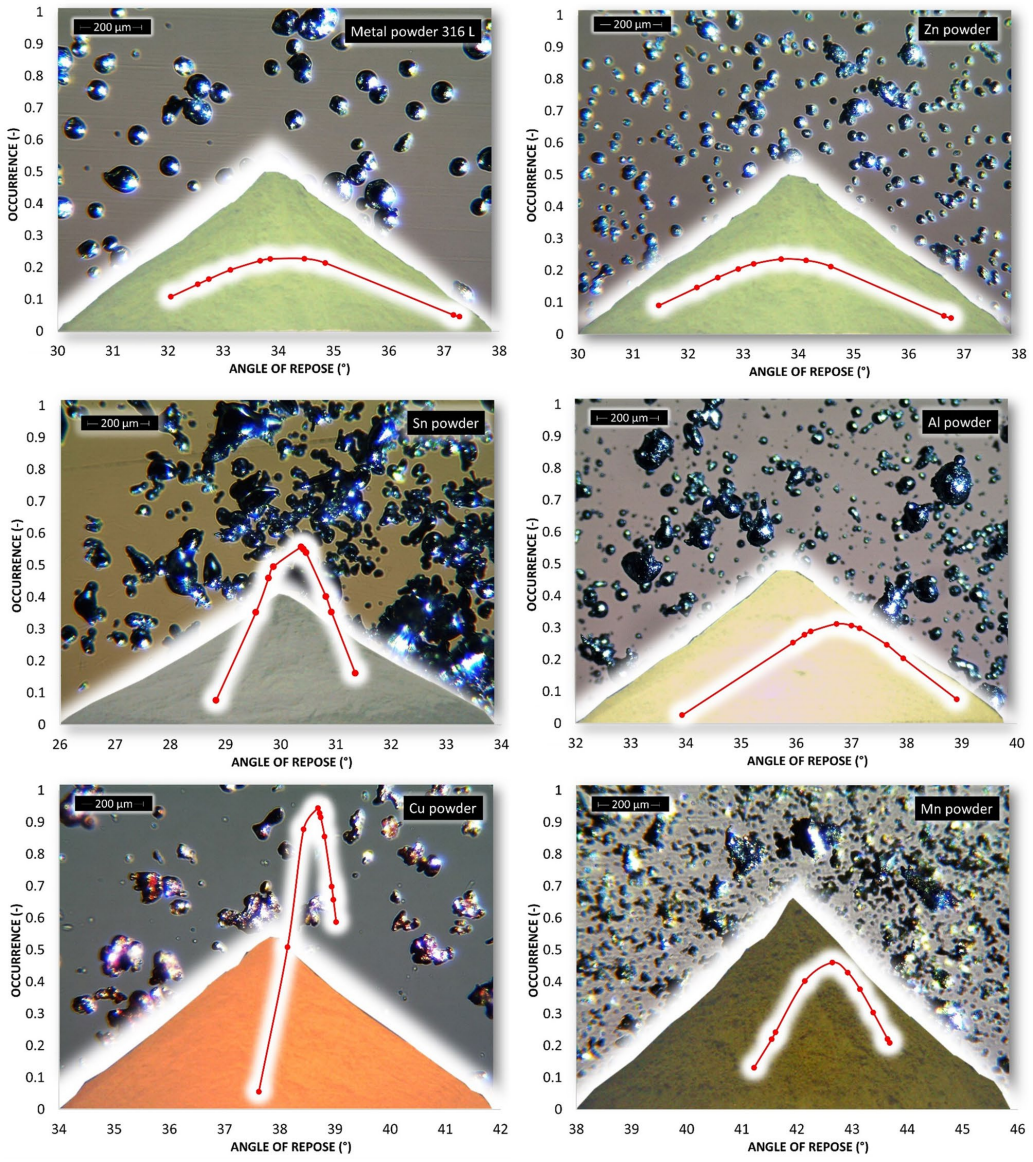
Occurrence of the static AOR for six metal powders.

**Figure 7:**
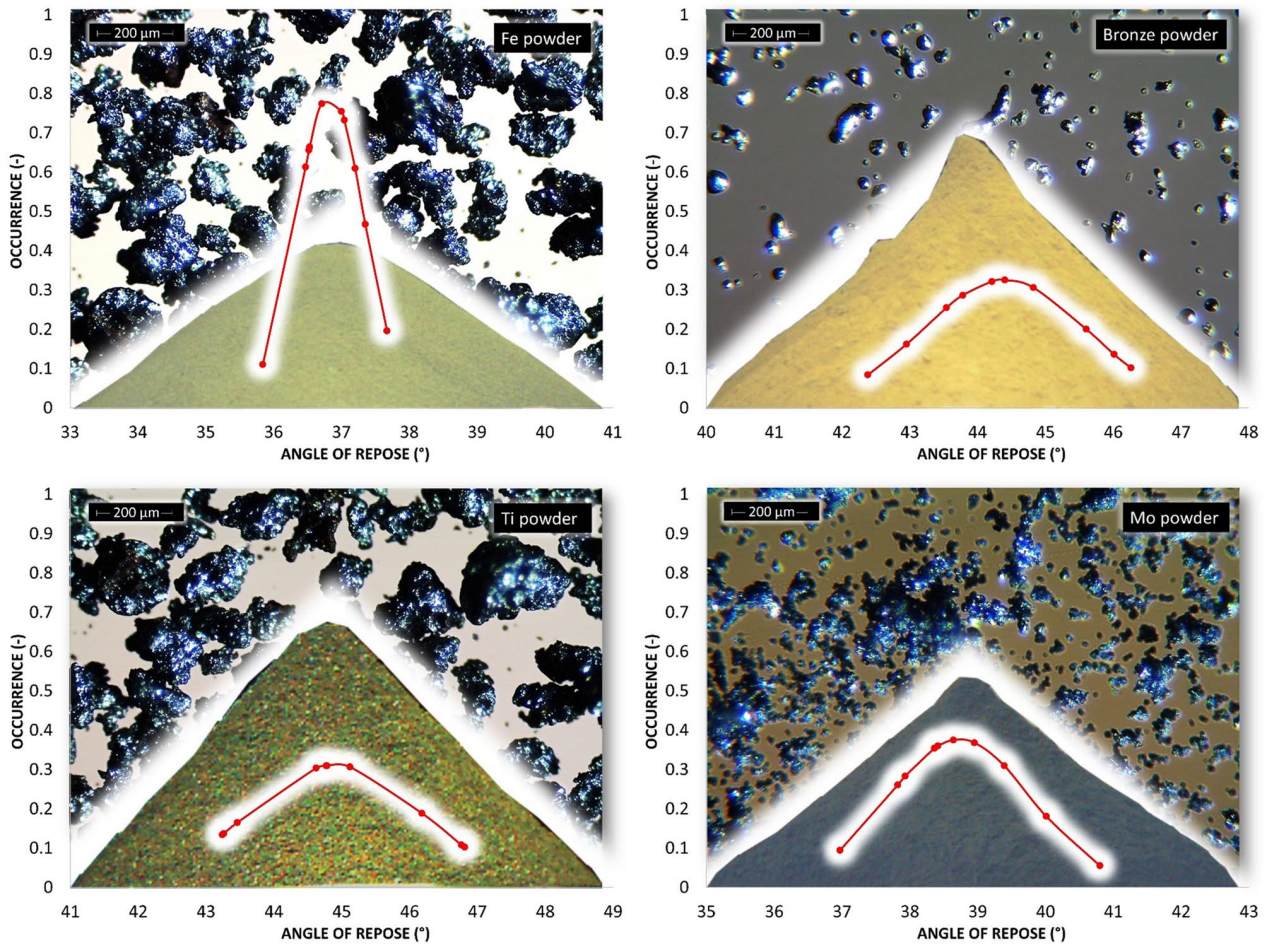
Occurrence of the static AOR for another four metal powders.

According to the parameters given in Table 2 and Fig. 5, titanium powder, whose d90 is equal to 452 μm, has the largest particle size. Other metal powders reach a maximum of 200 μm for parameter d90. The smallest particles are contained in zinc powder. Span S indicates the breadth of the particle size distribution. The most prominent S is for manganese powder (Table 2 and Fig. 5). A symmetrical particle size distribution is evident in zinc powder and iron powder. Microscopic and SEM photographs shown in the background of graphs of the occurrence of the static AOR, resp. AIFE (Figs. 6, 7, 10, 11, respectively) suitably supplement all the above-mentioned information. For example, titanium powder contains larger particles, some dendritic in shape (Fig. 12), manganese powder contains sharp-edged particles of various sizes (S = 2.5) (Fig. 11). In the case of manganese powder, the gaps between the larger particles can be filled with smaller ones (possibility of increase in packing density/packing fraction). Stainless steel powder contains separated particles with a shape very close to spherical. Tin powder is characterized by particles that look like ellipsoids (Fig. 11). Bronze powder is composed of a mixture of shapes from less regular to regular. Span can also be one of the parameters that minutely characterize the flowability of metal powders^44,51^. The limit value is considered to be S = 1.5. Powders having an S below this limit value show good flow properties (group 1), while powders with an S value higher than 1.5 (group 2) show rather worse flow properties (S ≫ 1.5). It is clear from Table 2 that Metal powder 316L, Zn powder, Cu powder, Fe powder and Ti powder should show excellent flowability. The other five metal powders would then be assigned to group 2 with poorer flow properties. This evaluation is only indicative, and it is necessary to evaluate the flow also using other methods listed below.

### Flowability of metal powders

#### Static AOR (Zenegero)

The evaluation of the flow properties of 10 samples of metal powders according to the AOR together with the values is given in Table 3. AOR is the average value from ten measurements, each of which contained an evaluation of 8 angles. The formed powder cones of all samples are shown in Figs. 6 and 7.

**Table 3:** Static AOR with standard deviation of measurement (σ_sd_) and classification of flow properties according to Table 1 for individual metal powders.

	AOR (°)	σ_sd_ (°)	Flow properties
Metal powder 316 L	34.2	2.0	Free flowing
Zn powder	33.8	2.0	Free flowing
Sn powder	30.2	1.4	Free flowing
Al powder	36.8	1.5	Free flowing
Cu powder	38.6	0.9	Fair to passable flow
Mn powder	42.6	1.4	Fair to passable flow
Fe powder	36.8	1.0	Free flowing
Bronze powder	44.4	1.8	Fair to passable flow
Ti powder	44.9	1.6	Fair to passable flow
Mo powder	38.7	1.9	Fair to passable flow

According to Table 1, the flow character was also inserted into Table 3. The tested metal powders can be divided into two groups, where 5 powders fall into the group with very good flow properties and the other group 5 powders into the group of a suitable, average flow mode. A prerequisite for a group of metal powders falling into the free-flowing mode appears to be a spherical or almost spherical shape of particles. In the case of iron particles with a rougher surface, with a size of up to about 200 µm, this also relates to an approximately spherical shape. The second group, characterized by a passable, average flow, contains particles with a wider particle size distribution, where a larger number of mutual contacts is possible. A tighter arrangement was therefore created by pouring into a cone formation (Fig. 7—Ti powder, Fig. 6—Mn powder). For samples with sharp-edged particles and more elongated shapes (Cu, Mn and Bronze powder), it is evident that the value of the AOR increases with their decreasing mean particle size. Higher values of AOR (Bronze, Ti and Mo powder) also indirectly indicate increased AIFE and mutual blocking of particles.

However, it can be stated that it is not yet possible to provide a general dependence (function) between the primary properties of metal powder particles (size, particle shape, surface structure) and flow properties according to AOR. Quantification of flow based on AOR does not provide sufficient conclusions. To obtain more accurate conclusions, the other methods below must also be used.

#### Dynamic AOR

**Figure 8:**
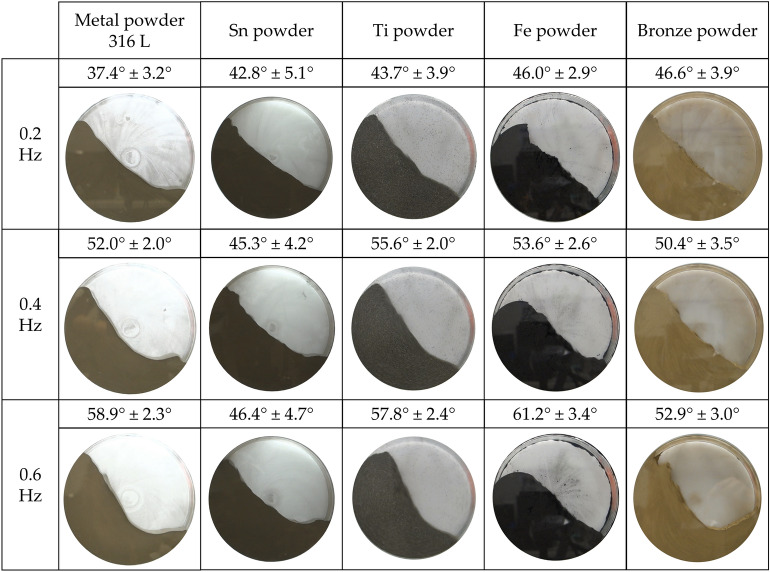
DAOR for first five metal powders.

**Figure 9:**
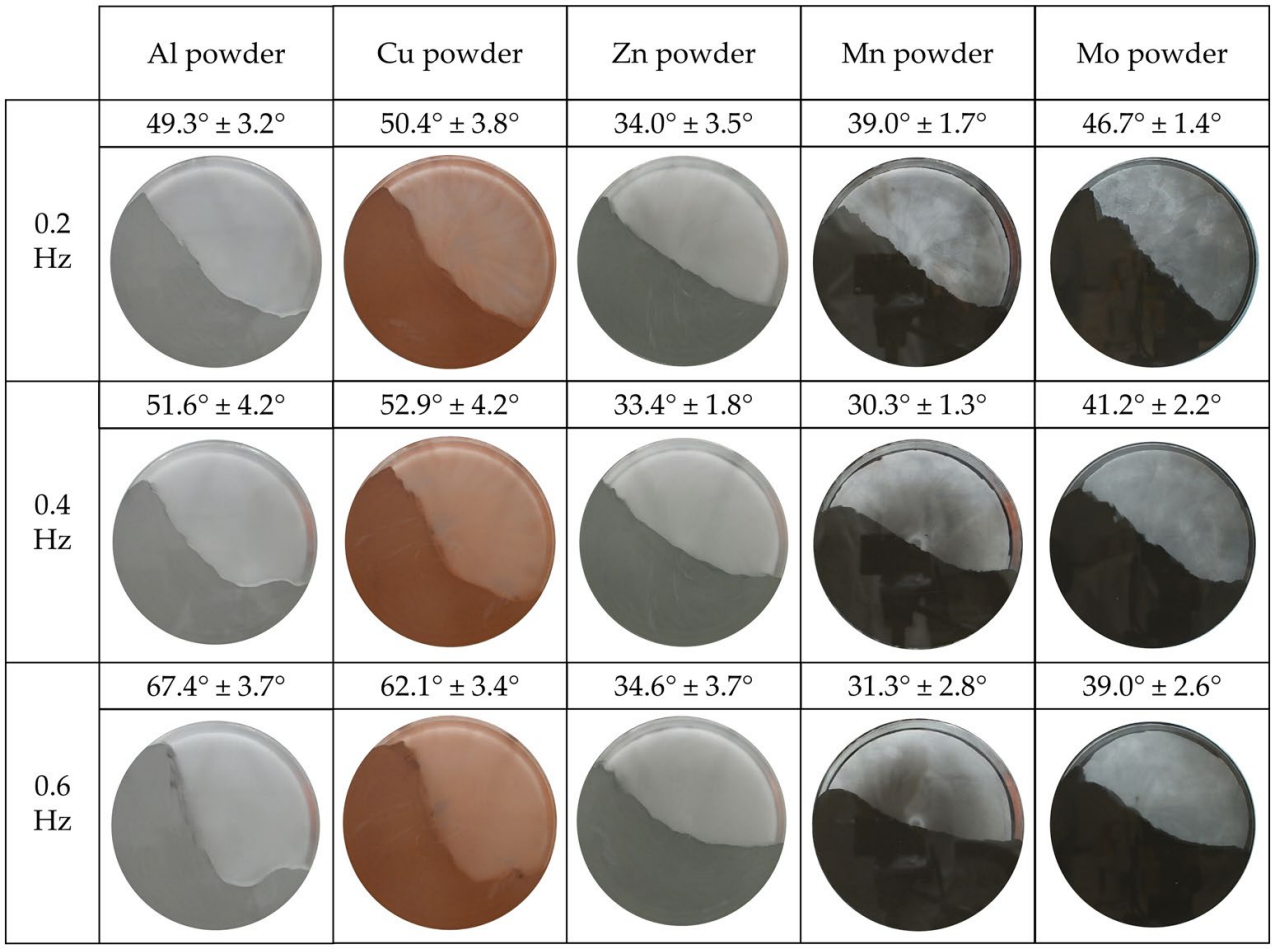
DAOR for another five metal powders.

Figures 8 and 9 provide a summary of dynamic angle of repose (DAOR) measurements. Most of the samples showed the character (hereinafter character 1) of increasing average values of measured angles with increasing rotational frequency from 0.2 to 0.6 Hz. Figure 8 shows all samples, i.e. metal powder 316L, Sn, Ti, Fe and Bronze powder, and in Fig. 9 it is Al and Cu powder.

The largest total differences in the values ​​of average angles for rotational speeds from 0.2 to 0.6 Hz were measured for the Metal powder 316 L sample. A change in the flow character could also be observed with increasing rotation frequency, from a rolling motion at 0.2 Hz to a cascading motion at 0.6 Hz. The average value of the angle from the three partial velocities was with a high standard deviation, namely 49.4° ± 9°. The smallest difference in the values ​​of the average angles for character 1 materials was found for the Sn powder sample. The average value of the angles had the smallest deviation, being 44.8° ± 1.5°. Thus, the best overall dynamic flow stability, therefore, was determined for Sn powder, for character 1 materials. By observing the nature of the flow during the measurement of the DAOR, stability can also be confirmed in a constant rolling motion at all tested frequencies. The character of the flow changed most significantly with increasing frequency of Al powder. At lower frequencies, Al powder showed rolling motion and, gradually, even a cataracting motion appeared.

The Mo powder material (sample) (Fig. 9) did not meet the character 1 criteria at all. The phenomenon was rather of the opposite nature and was hereinafter referred to as "character 2". The total average value of the angle from the set speeds was 42.3° ± 3.2°. The deviation was neither very low nor too high in relation to the other samples.

The smallest changes in the measured angles for the individual speeds from 0.2 to 0.6 Hz from all samples were displayed by the sample of Zn powder, which had an average value from three rotation speeds of 34° ± 0.5°. Due to the low deviation, this stability of the angle values with the change of the rotational frequency was called character 3.

The Mn powder sample fell into the category of character 2, with an overall average angle value of 33.5° ± 3.9°after the change in frequency. This was mainly due to the angle deviation values.

In the case where the individual velocities are assessed separately, for the frequency of 0.2 Hz the smallest deviation for the Mo powder sample was 46.7° ± 1.4°. Largest for Sn powder 42.8° ± 5.1°. For 0.4 Hz, the smallest deviation for Mn powder was 30.3° ± 1.3° and the largest for Sn powder was 45.3° ± 4.2°. For 0.6 Hz, the smallest deviation for Metal powder 316 L was 58.9° ± 2.3° and the largest deviation for Sn powder was 46.4° ± 4.7°.

Though the Sn powder sample came out as the most stable in character 1 overall from a dynamic standpoint, it had the highest separate angle deviations for individual rotation frequencies. The lowest angle deviations came at a speed of 0.2 Hz for Mo and Mn powder. At a frequency of 0.4 Hz, it was for Mn and Zn powders. Finally, at 0.6 Hz for Metal powder 316 L and Ti powder. At 0.6 Hz, Mo powder was in third place and Mn powder in fourth. From the measured data, it can be concluded that materials such as Mo and Mn powder show good dynamic stability at partial frequencies of the drum used. Although the Sn powder sample showed the largest deviations in terms of overall dynamic behavior at partial rotational frequencies, it also showed good angle stability.

From the overall point of view of stability in the dynamic process, one sample of "character 3" (very stable) was found, and that was Zn powder. The stability of the Zn flow dynamics can also be observed from the rolling motion shown, which occurred at all frequencies tested (Fig. 9). The second most stable material was found to be Sn powder with a character of 1. Next in line are Mn powder and Mo powder.

The greatest occurrence of the smallest particles from all d90 samples was measured for Zn powder. The question is how this property can affect the ductility of the material level during the dynamic flow in the rotating cylinder of the dimensions used. Other materials such as Sn powder, Mn powder and Mo powder also had a d90 of up to 100 µm. In terms of dynamic behavior in a rotating cylinder, these materials are closest to each other with their behavior (stability). Meanwhile, the flow indexes *ffc* and the values of the AOR flow angles separate Zn and Sn powder from Mn and Mo powders according to their evaluation (Table 5, Free-flowing vs. Easy-flowing).

In terms of angle values, the smallest angle turned out to be at a rate of 0.2 Hz for the Zn powder sample and Metal powder 316 L. The largest angle was then for Cu and Al powder. For 0.4 Hz, the smallest angle was for Mn, Zn powders and the largest for Ti, Cu powders. For 0.6 Hz, the smallest angle was for Mn, Zn powders and the largest for Al, Cu powders. The Zn powder sample appears among the smallest values most often. In terms of flow and AOR of selected samples, Zn powder is among those with the best flow properties.

#### Shear cell procedures—AIFE and flow index

**Figure 10:**
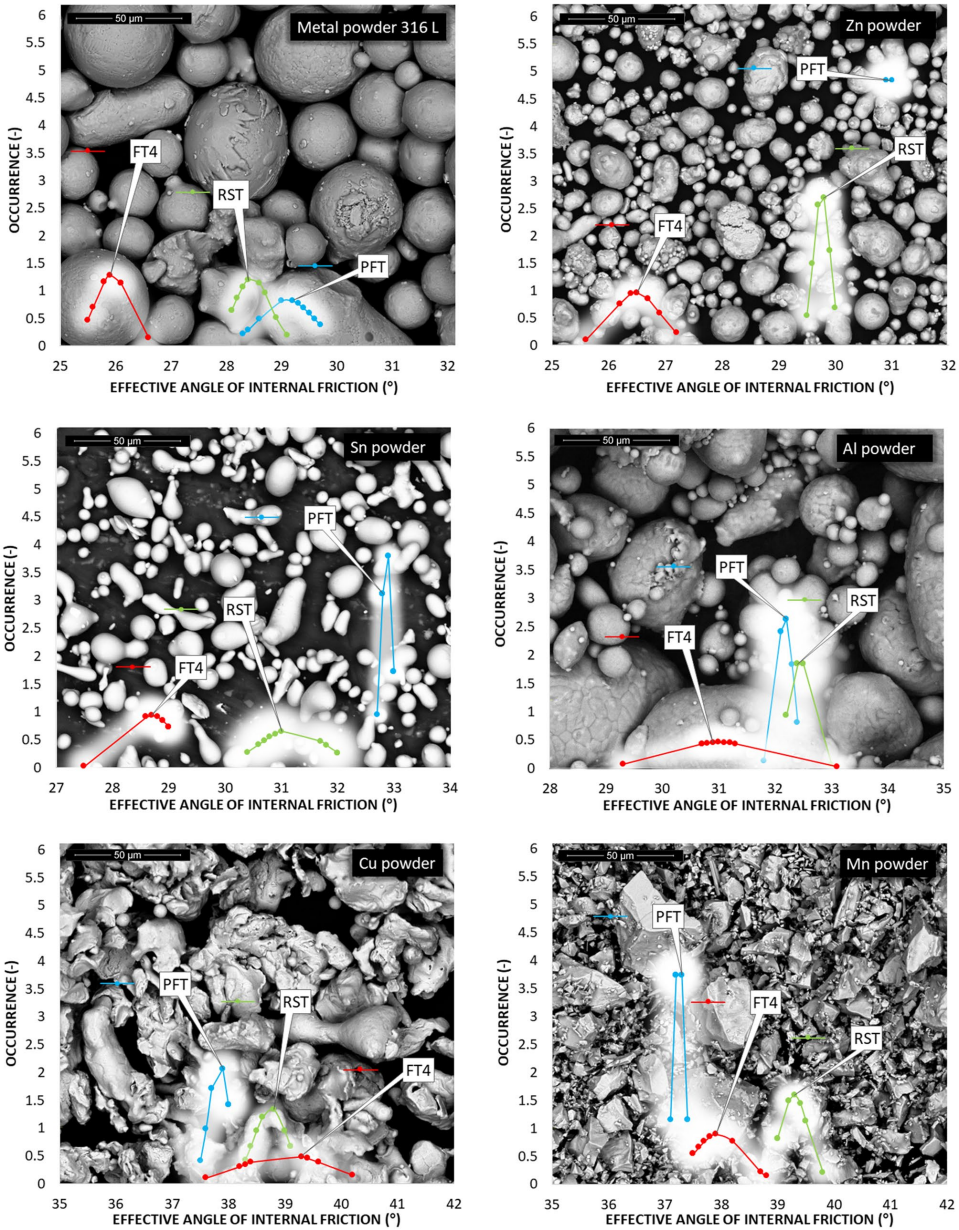
Occurrence of the AIFE for first six metal powders.

**Figure 11:**
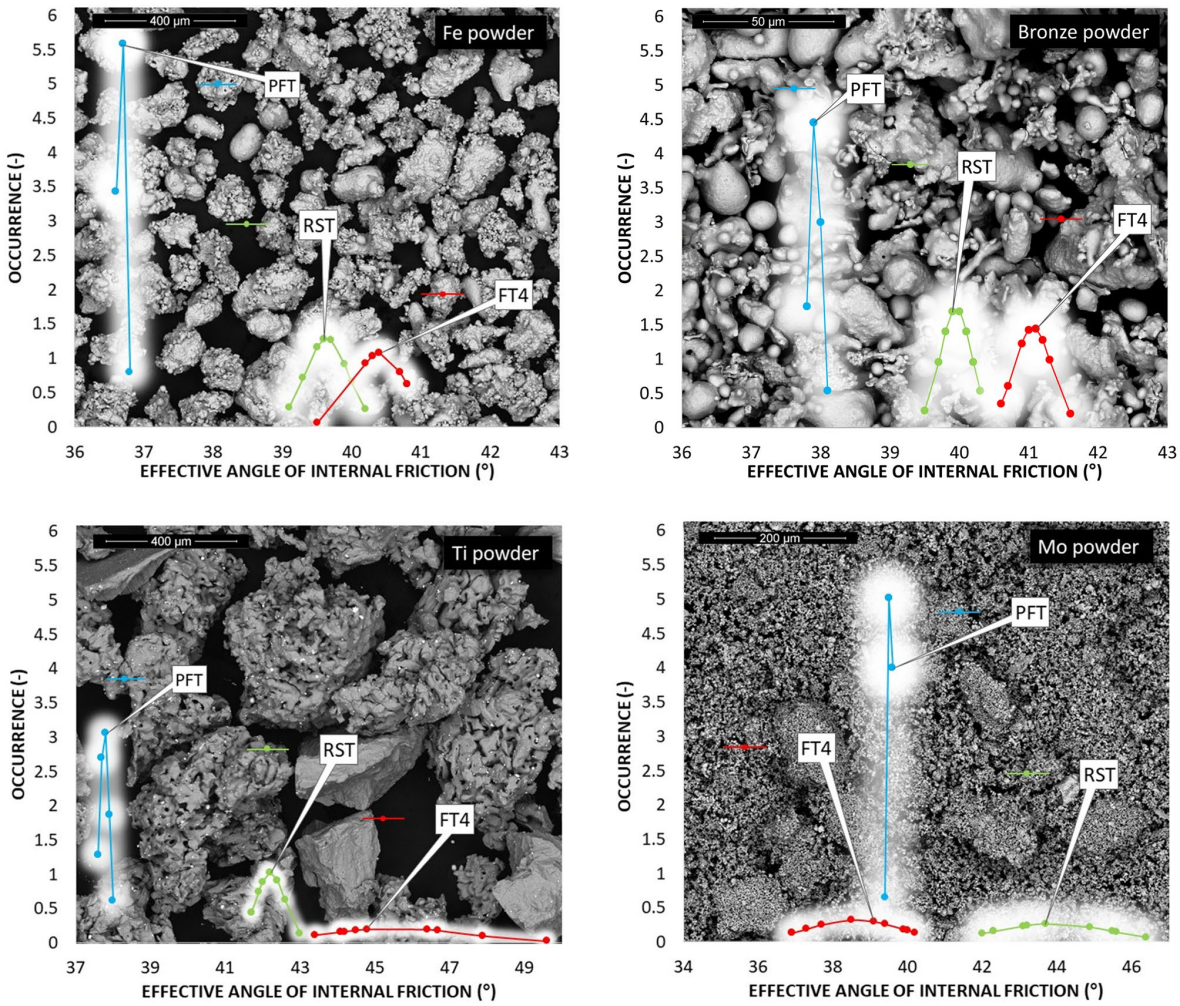
Occurrence of the AIFE for another four metal powders.

The AIFE were determined based on three commonly used devices: RST, PFT and FT4. The resulting data are shown in Figs. 10, 11 and Table 4.

**Table 4:** AIFE with standard deviation of measurement for three shear ring devices.

	RST		PFT		FT4	
	AIFE (°)	σ_sd_ (°)	AIFE (°)	σ_sd_ (°)	AIFE (°)	σ_sd_ (°)
Metal powder 316 L	28.5	0.3	29.1	0.5	26.0	0.3
Zn powder	29.8	0.1	31.0	0.1	26.5	0.4
Sn powder	31.2	0.6	32.9	0.1	28.7	0.4
Al powder	32.5	0.2	32.2	0.1	31.0	0.9
Cu powder	38.8	0.3	37.8	0.2	39.0	0.8
Mn powder	39.3	0.3	37.3	0.1	38.0	0.4
Fe powder	39.6	0.3	36.7	0.1	40.4	0.4
Bronze powder	40.0	0.2	37.9	0.1	41.1	0.3
Ti powder	42.2	0.4	37.8	0.1	45.6	1.9
Mo powder	43.9	1.5	39.5	0.1	38.6	1.2

Figures 10 and 11 show the ranges of measured values from RST, PFT and FT4. In all cases of metal powder tests, except aluminum, the range of absolute values was the lowest in the case of the PFT machine. This is probably due to the methodology of the evaluation software. The largest (most pronounced) variations in the values of effective angles of AIFE for metal powders were achieved in the results from the FT4 machine. In the case of aluminum powder, this considerable breadth can probably be explained by the fact that it is a light soft metal and its (plastic) deformation can easily occur. In the case of titanium powder, which contains 90% of particles up to 452 µm, it could be concluded that the considerable variance of the values of effective angles is given precisely by the size and shape of the particles. During the measurement, larger titanium particles are wedged into the space between the glass cylinder and the measuring impeller (shear head). Although this space has a size of 1 mm (Fig. 4), the shape of the titanium particles allows such an arrangement that causes an increase in the resistance of the impeller to the direction of movement and consequently also an increase in shear stress.

**Figure 12:**
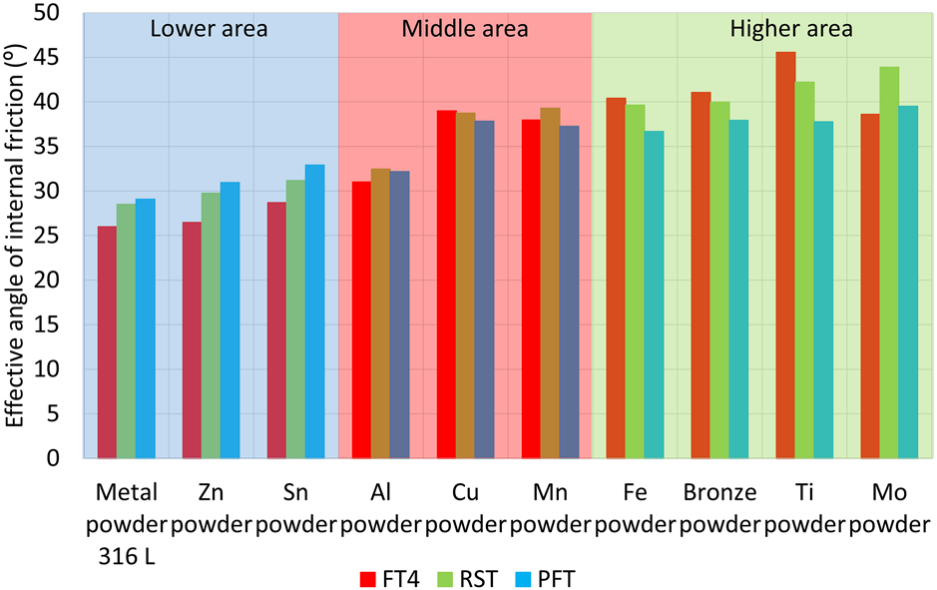
Comparison of AIFE between FT4, RST, PFT for tested metal powder.

Figures 11 and 12 also show an SEM image for the AIFE from RST, PFT and FT4. Spherical, oval and smooth shapes were most pronounced in the samples Metal powder 316 L, Zn, Sn, Al (Fig. 11). These shapes had a major effect on the values of the AIFE, which fell into the Lower Area (316L, Zn). Figure 12 shows the samples which, due to their grain shapes, fell into the Higher Area in terms of the values of the AIFE. Higher values of the AIFE in the examined samples were influenced by the complexity of the shape of individual particles.

The tested metal powders were divided into three groups—lower, middle and top area (Fig. 12), according to the corresponding specific values of the AIFE. The lower area corresponds to the values of the lowest measured values of the AIFE (Metal powder 316L, Zn powder, Sn), the middle area to values in the range of approximately 32°–37° (Al, Cu and Mn), the last higher area then to all higher angles (Fe, Bronze, Ti and Mo).

It can be seen from Fig. 12 that the smallest differences between the measured values of AIFE are in the middle area. The largest difference, i.e. 2°, in this area is between RST and PFT for the Mn powder sample. In the case of the group of metal powders located in the lower area, the powder bed loosens on FT4 (standardization) of sample preparation during the actual measurement (part of the measurement). The impeller of this device penetrates through the entire powder bed of the sample and the test powder is loosened and the sample treatment is unified before the actual measurement of the dependence of shear friction on the normal load. Thus, FT4 probably shows smaller values of the AIFE due to the loosening (aeration) step, when the largest difference of 4.5° in the Zn powder sample is between FT4 and PFT. For the higher area, it is evident (Fig. 12) that the largest difference of 7.8° is for the Ti powder sample, again between the FT4 and PFT machines. Unlike the lower area, however, in the higher area, FT4 shows higher values than PFT. This phenomenon is probably due to the design differences of the cells used (Fig. 4). Based on the differences in the results of AIFE determined on different devices, it is necessary to pay attention to the accuracy of the measured results according to the deviation of the measured values (see: Gaussian waveforms in Figs. 10 and 11) and during the measuring, lean toward the machines that show the smallest differences in the average values from the measurements between each other. In this middle area, it can be seen (Fig. 12) that for the Al powder material this is a difference of 0.3° between the PFT–RST, for the Cu powder material it is FT4–RST with a difference of 0.3° and for the Mn powder material it is FT4–PFT with a difference of 0.7°.

The large variance of the values in Fig. 11 for the titanium powder indicates that FT4 is less suitable for this material than PFT and RST. This is similar in the case of the RST machine for Mo powder. The calculated Mo powder span is unusually large compared to the values of almost all other samples (Table 2). Another practical outcome is the fact that, in the peripheral areas, between the machines used, the RST machine shows the mean (average) values of AIFE and the Brookfield machine has the most stable and smallest deviations in measurement.

#### Shear cell procedures—flowability

The flow index *ffc* (flowability) is the relationship between the unconfined yield strength and the major principal stress during consolidation (Fig. 1). Using this parameter, it is possible to classify metal materials into individual flow modes (Fig. 1). The *ffc* values set by all three devices used are listed in Table 5.

**Table 5:** Flow index *ffc* (–) for three shear ring devices.

	RST		PFT		FT4	
	ffc (–)	Flow	ffc (–)	Flow	ffc (–)	Flow
Metal p. 316 L	71	Free-flowing	15	Free-flowing	13	Free-flowing
Zn powder	81		16		29	
Sn powder	> 100		28		15	
Al powder	> 100		43		15	
Cu powder	24		14		9	Easy-flowing
Mn powder	24		8	Easy-flowing	7	
Fe powder	46		> 100	Free-flowing	19	Free-flowing
Bronze powder	17		5	Easy-flowing	5	Easy-flowing
Ti powder	24		37	Free-flowing	10	
Mo powder	15		5	Easy-flowing	6	

According to the *ffc* flow index, the Schulze device characterized all tested powders as free-flowing. The *ffc* flow modes are then very similar for the PFT and FT4. They differ only in the case of Cu and Ti, where these are limit values for FT4. However, it is evident that the sensitivity of the flow index itself to be included in flow regimes is not entirely sufficient.

As can be seen, according to *ffc*, Mn powder, Bronze powder, and also Mo powder, belong to the easy-flowing group, i.e. to a regime that is having a flow rate that is worse by a degree than all other tested metal powders. Mn, Mo, and also Bronze powder, contain relatively small particles (d10 is up to 12 μm). In general, particle size is one of the dominant properties for flow^52–55^. Reducing the particle size can lead to a reduction in flow as the surface area of the particles increases, the surface area for the interaction of surface cohesive forces increases, leading to a more cohesive flow. Interparticle forces are more significant compared to particle weight. However, as can be seen from the results, powders with very similar particle sizes may show different flow behaviors. Zn and Sn powders contain particles whose d10 is also up to a maximum of 12 μm. Considering their flow, other properties are probably already dominating here. These are probably the morphology and surface roughness of the particles. Sn and Zn contain spherical, smooth particles. The flow regime is therefore free-flowing for these powders. The Mo, Mn and Bronze discussed above contain particles with a rough surface, and irregular shapes, which placed their flowability in the area of easy-flowing. Unusually high *ffc* values (Sn, Al powder) are probably result from the fact that the set normal tension is too small for these materials. It can therefore be attributed to the machine settings and it must be mentioned that in these cases the deformation of the particles can also have an effect.

In most cases, the lowest *ffc* values are obtained for the FT4. The exceptions are Zn and Mo powder. The *ffc* values are probably related to the size of the slip surface, which is the largest in RST (8482 mm^2^), then for the PFT (4750 mm^2^) and finally the FT4 (1879 mm^2^). In general, bulk materials flow better through a larger cross-section.

If the same normal loads were used for the RST, PFT and FT4 measurements, and *ffc* came out differently, as a rule, different σ_c_ strengths had to be achieved. The highest σ_c_ unconfined yield strength was achieved with the FT4. Since the largest cell volume was in RST and the smallest in PFT, the σ_c_ strength had to be related to the material height, which was highest in FT4 (44.5 mm), followed by RST (19.3 mm), and PFT (9 mm). Thus, a smaller shear cross section and a higher column of material increase the overall σ_c_ strength and thus reduce the flowability.

**Figure 13:**
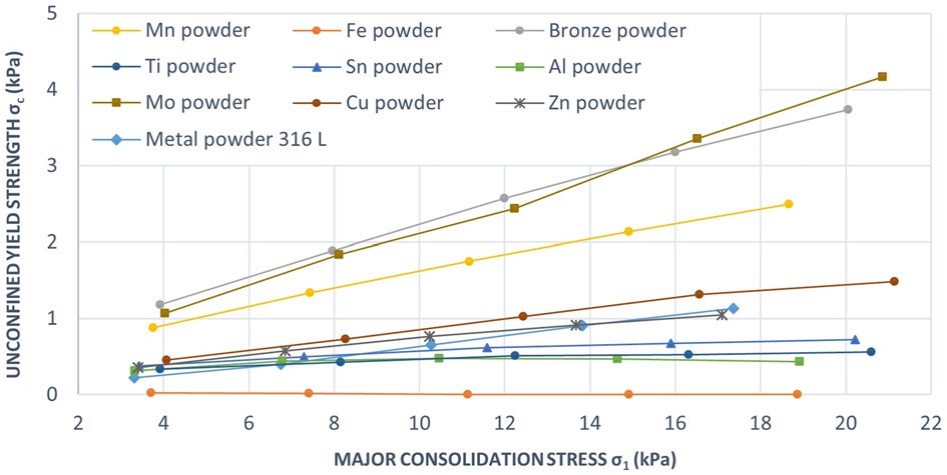
Dependence unconfined yield strength on major principal stress.

To clarify the course of the flow function, the Fig. 13 is given. The dependence σ_c_ on σ_1_ was obtained from PFT shear tester for every powder. All metal powders were tested at normal load 2, 4, 6, 8 and 10 kPa.

For almost metal powders, σ_c_ increases with increasing σ_1_. The slope of this linear dependence is higher for Bronze powder, Mo and Mn powders than slope of the lines of Cu, Zn, Sn Al, Ti powders and Metal powder 316 L. A higher value of the linear slope indicates a flow resistance. For Fe powder the slope of the line corresponds to a very low value close to zero, which is an indication of free-flowing powder.

### Results comparison

A comparison of the results, where the flowability of metal powders is characterized even using the various techniques presented in this paper, is given in Table 6.

**Table 6:** Metal powder flow evaluation according to different testing methods.

	Flow according to Span S	Flow according to AOR	Character according to DAOR	Area according to AIFE	Flow according to ffc (FT4)
Metal powder 316 L	Good flow	Free flowing	1	Lower	Free flowing
Zn powder	Good flow	Free flowing	3	Lower	Free flowing
Sn powder	Passable flow	Free flowing	1	Middle	Free flowing
Al powder	Passable flow	Free flowing	1	Middle	Free flowing
Cu powder	Good flow	Fair to passable flow	1	Middle	Easy-flowing
Mn powder	Passable flow	Fair to passable flow	2	Middle	Easy-flowing
Fe powder	Good flow	Free flowing	1	Higher	Free-flowing
Bronze powder	Passable flow	Fair to passable flow	1	Higher	Easy-flowing
Ti powder	Good flow	Fair to passable flow	1	Higher	Easy-flowing
Mo powder	Passable flow	Fair to passable flow	2	Higher	Easy-flowing

**Figure 14:**
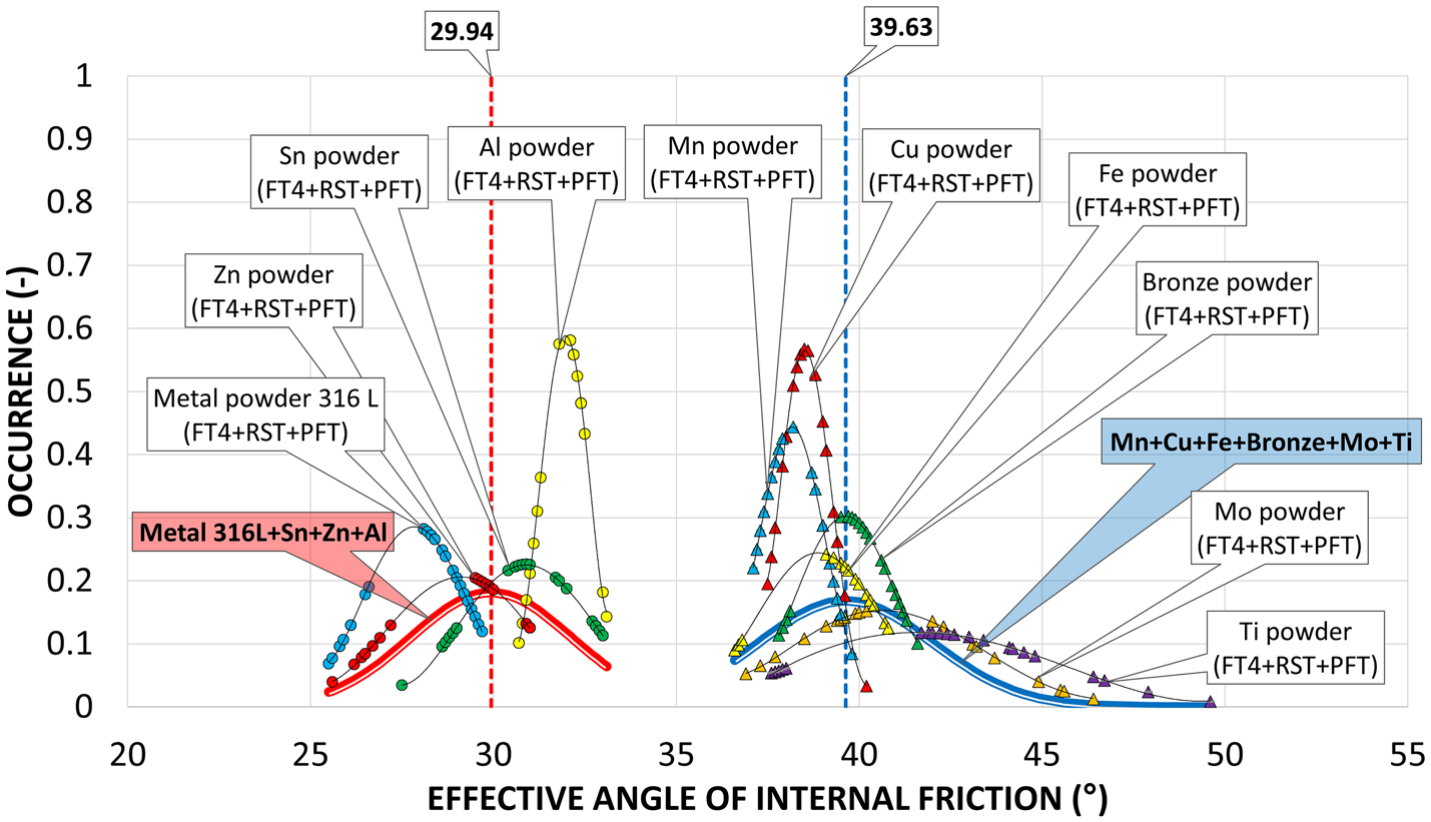
Comparison of AIFE combine values for FT4 + RST + PFT for tested metal powders, combining the materials into two groups.

Another comparison of the results concerns the relationships between the AIFE values and AOR. In the comparison of dependencies between the measured values of AOR and AIFE, values were measured from three shear devices (FT4, RST, PFT), combined into one set of values for each metal material, and shown using Gaussian waves on the graph in Fig. 14.

**Figure 15:**
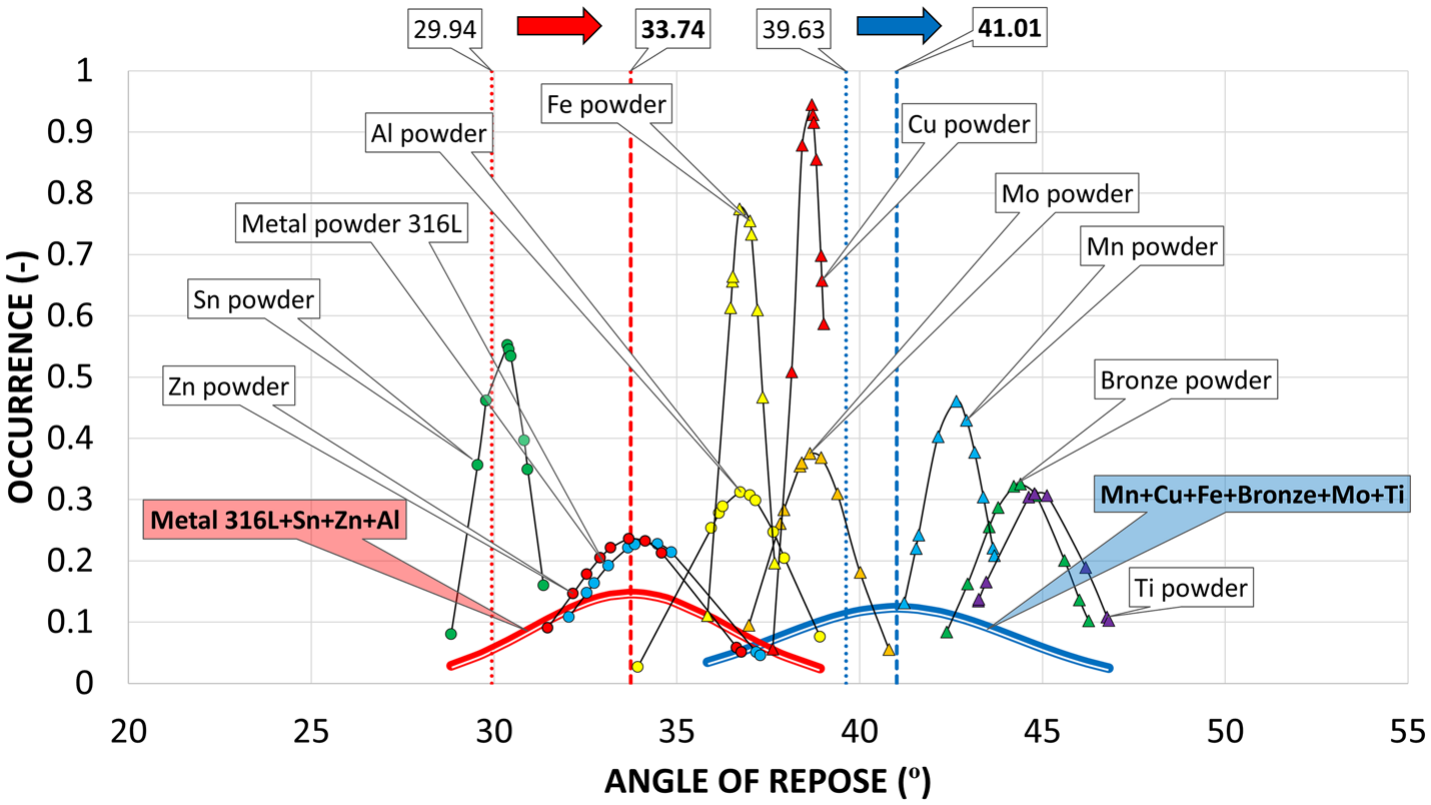
Comparison AOR for tested metal powders with a comparison of groups material occurrence between the AOR and the AIFE.

It can be seen on the graph that the measured materials created 2 areas where the measured values of the individual materials cluster. According to these areas, one group of materials was combined into a red Gaussian curve (Metal powder 316L, Sn powder, Zn powder, Al powder) and the other group of materials into a blue Gaussian curve (Mn powder, Cu powder, Fe powder, Bronze powder, Mo powder, Ti powder). These two groups of equally represented metallic materials were also created for comparison in the graph of measured values of the AOR (Fig. 15).

Since the general relationship between the primary properties of metal powder particles (size, particle shape, surface texture) and flow properties according to AOR is not yet sufficiently known, it is unnecessary to compare individual measured values of AIFE at which the material is loaded with normal force and the AOR, where the material is loosely poured without normal load. When comparing how groups of materials behave on the angular axis, it is possible to notice the conversion patterns, between the AOR and the AIFE. The first group of materials shown in the graph of AIFE by a red Gaussian curve with an average value of 29.9° ± 2.2° occurs for the same group of materials in the graph of measured values of AOR shifted to the right with an average value of 33.74° ± 2.72°, i.e., with a difference of 3.8°.

The second group shown in the graph of AIFE by a blue Gaussian curve with an average value of 39.6° ± 2.4° occurs for the same group of materials in the graph of measured values of AOR also shifted to the right with an average value of 41.01° ± 2.4°, but with a smaller difference of 1.41°. From the evaluation, it can be deduced that larger differences between the measured values of AOR and AIFE occur in the area of the AOR classification of Very Free-flowing and Free-flowing and smaller differences occur in the modes Fair to passable flow and Cohesive (Table 6).

## Conclusion

The aim of the paper was to evaluate the flow properties of metal powders and to conduct research in terms of possible dependences of mechanical-physical properties of mixtures, particles on flow characteristics and other parameters. Several conventional methods were used for the assessment, which are based on known classifications of bulk materials and are still current and used methods, for example in the field of digitization or Industry 4.0.

The research of metal powders leads to the following fundamental conclusions:

1. Half of the samples showed values of Span below 1.5 (Metal powder 316 L, Zn, Cu, Fe, Bronze and Ti powder), i.e., excellent flow properties. The rest of the samples had worse flow properties, but still acceptable in terms of the overall range of powder materials.
2. The classification according to the AOR also divided the group of samples into two groups. These were Free flowing (50% of samples) and Fair to passable flow. The lowest AOR value was measured for Sn powder, 30.2°, and the highest for Ti powder at 44.9°. A possible dependence of the AIFE on AOR was found, which was supported by the shape of individual particles shown in the SEM photographs.
3. In terms of dynamic AOR, lower angles were measured for samples with lower values of AIFE and higher *ffc* values. Three characteristics of flow stability were determined while changing drum rotation frequency. Character 1 had an increasing tendency of angle values with increasing drum frequency. This included all samples except Zn, Mn and Mo powders. Character 2 had the opposite effect. This included Mo powder and partly Mn powder. Character 3 was manifested by a minimal change in the angle when changing the frequency, namely in the case of Zn powder, which was one of the better flowing in terms of *ffc* and the AIFE.
4. In most cases, the lowest *ffc* values were obtained for FT4. The exceptions were Zn and Mo powders. The *ffc* values may be related to the size of the shear surface, with the largest being for the RST (8482 mm2), then the PFT (4750 mm2) and finally the FT4 (1879 mm2). In general, bulk materials flow better through a larger cross-section.

The samples were divided into three groups (Lower, Middle and High area), according to the AIFE. In the lower area group, the lowest values were measured for FT4, then for RST, and finally for PFT. In the high area group, it was rather the opposite. Evidence has not yet been obtained that this was a property of the cell construction or of the tested samples. However, the inverse character could however be crucial in the field of force design according to Janssen's equations, which use the values of effective angles, especially in applications with materials having worse flowability.

An SEM image was intentionally assigned to the AIFE of RST, PFT and FT4. Spherical, oval, and smooth shapes were most pronounced in the samples of Metal powder 316 L, Zn, Sn, and Al. These shapes had a major effect on samples with an AIFE, which fell into the Lower Area (316L, Zn, Sn,). Higher values of the AIFE in the examined samples were influenced by the complexity of the shape of individual particles.

5. The results achieved show that the individual samples required an individual approach in terms of accurate determination of flow characteristics. Similar flow characteristics were found in different assessment methods. The predominant spherical shape of individual particles in the samples Metal powder 316 L, Zn, Sn and Al powder was proved as the basic input parameter having a fundamental influence on flow characteristics, which is also evident from the evaluation of the measurement of the AIFE, which is shown in Fig. 14.
6. All the evaluations presented in this paper deepen the understanding of the behavior of bulk material and contribute significantly to finding general dependencies in metallic powder materials. By comparing different metal powders, equipment, measuring methods, it is possible to gradually reveal the laws of bulk materials, and use them in 3D printing with metal powders and other applications.

## List of symbols

AIFE: Effective angle of internal friction (°)
AOR: Angle of repose (°)
c: Cohesion (Pa)
d_10_: 10% Particle representation for the detected size, cumulative phase lower decile (µm)
d_50_: 50% Particle representation for the detected size, median (µm)
d_90_: 90% Particle representation for the detected size, cumulative phase upper decile (µm)
DAOR: Dynamic angle of repose (°)
ffc: Flow index (–)
S: Span (–)

## Greek symbols

σ_1_: Major consolidation stress (Pa)
σ_c_: Unconfined yield strength (Pa)
σ_sd_: Standard deviation (°)
ϕ: Linearized angle of internal friction (°)

## Abbreviations

Al: Aluminium powder
AM: Additive manufacturing
BJ: Binder jetting
Cu: Copper powder
EPBF: Electron-beam powder-bed fusion
Fe: Iron powder
FT4: Powder rheometer FT4
LPF: Laser powder-fed
Mn: Manganese powder
Mo: Molybdenum powder
PFT: Brookfield Powder Flow Tester
RST: Schulze ring shear tester RST-01.pc
SEM: Scanning electron microscope
SLM: Selective laser melting
Sn: Tin powder
Ti: Titanium powder
Zn: Zinc powder

## References

[1] Bremen S, Meiners W, Diatlov A (2012). Selective laser melting. Laser Tech. J..

[2] Alimardani M, Toyserkani E, Huissoon JP, Paul CP (2009). On the delamination and crack formation in a thin wall fabricated using laser solid freeform fabrication process: an experimental-numerical investigation. Opt. Lasers Eng..

[3] Basalah A, Shanjani Y, Esmaeili S, Toyserkani E (2012). Characterizations of additive manufactured porous titanium implants. J. Biomed. Mater. Res. Part B Appl. Biomater..

[4] Nandwana P (2017). Powder bed binder jet 3D printing of Inconel 718: densification, microstructural evolution and challenges☆. Curr. Opin. Solid State Mater. Sci..

[5] Segura IA (2019). Grain boundary and microstructure engineering of Inconel 690 cladding on stainless-steel 316L using electron-beam powder bed fusion additive manufacturing. J. Mater. Sci. Technol..

[6] Zhang Y (2018). Additive manufacturing of metallic materials: a review. J. Mater. Eng. Perform..

[7] Fayazfar H (2018). A critical review of powder-based additive manufacturing of ferrous alloys: process parameters, microstructure and mechanical properties. Mater. Des..

[8] Barletta, D., Poletto, M. & Santomaso, A. C. Chapter 4. Bulk powder flow characterisation techniques. In *Powder Flow* (eds Hare, C. et al.) (2019). 10.1039/9781788016100-00064.

[9] Wischeropp TM, Emmelmann C, Brandt M, Pateras A (2019). Measurement of actual powder layer height and packing density in a single layer in selective laser melting. Addit. Manuf..

[10] Frykholm R, Takeda Y, Andersson BG, Carlstrom R (2016). Solid state sintered 3-D printing component by using inkjet (binder) method. Funtai Oyobi Fummatsu Yakin/J. Jpn. Soc. Powder Powder Metall..

[11] German RM (1992). Prediction of sintered density for bimodal powder mixtures. Metall. Trans. A.

[12] Dourandish M, Godlinski D, Simchi A (2007). 3D printing of biocompatible PM-materials. Mater. Sci. Forum.

[13] Vasilenko A, Glasser BJ, Muzzio FJ (2011). Shear and flow behavior of pharmaceutical blends—method comparison study. Powder Technol..

[14] Prescott JK, Barnum RA (2000). On powder flowability. Pharm. Technol..

[15] MiDi GDR (2004). On dense granular flows. Eur. Phys. J. E.

[16] Pleass C, Jothi S (2018). Influence of powder characteristics and additive manufacturing process parameters on the microstructure and mechanical behaviour of Inconel 625 fabricated by Selective Laser Melting. Addit. Manuf..

[17] ASTM International. *Standard Test Method for Measuring the Angle of Repose of Free-Flowing Mold Powders*. *C 1444-00* (2000). 10.1520/C1444-00 (2000).

[18] Massaro Sousa L, Ferreira MC (2019). Densification behavior of dry spent coffee ground powders: experimental analysis and predictive methods. Powder Technol..

[19] Jenike, A. Storage and flow of solids, bulletin no. 123. *Utah Eng. Exp. Stn.* (1964).

[20] Beakawi Al-Hashemi HM, Baghabra Al-Amoudi OS (2018). A review on the angle of repose of granular materials. Powder Technol..

[21] Riley GS, Mann GR (1972). Effects of particle shape on angles of repose and bulk densities of a granular solid. Mater. Res. Bull..

[22] Fraczek J, Złobecki A, Zemanek J (2007). Assessment of angle of repose of granular plant material using computer image analysis. J. Food Eng..

[23] Rackl M, Grötsch FE (2018). 3D scans, angles of repose and bulk densities of 108 bulk material heaps. Sci. Data.

[24] Nedderman, R. M. *Statics and Kinematics of Granular Materials* (1992). 10.1017/cbo9780511600043.

[25] Kleinhans MG, Markies H, De Vet SJ, In’t Veld AC, Postema FN (2011). Static and dynamic angles of repose in loose granular materials under reduced gravity. J. Geophys. Res. E Planets.

[26] Nakashima H (2011). Determining the angle of repose of sand under low-gravity conditions using discrete element method. J. Terramech..

[27] Schwedes J (2003). Review on testers for measuring flow properties of bulk solids. Granul. Matter.

[28] Schulze, D., Schwedes, J. & Carson, J. W. *Powders and Bulk Solids: Behavior, Characterization, Storage and Flow* (2008). 10.1007/978-3-540-73768-1.

[29] Rhodes, M. *Introduction to Particle Technology: Second Edition*. *Introduction to Particle Technology: Second Edition* (Wiley, Chicester, 2008). 10.1002/9780470727102.

[30] Carson JW, Wilms H (2006). Development of an international standard for shear testing. Powder Technol..

[31] ASTM International. D6773-02: Standard Test Method for Bulk Solids Using Schulze Ring Shear Tester 1. *Annu. B. ASTM Stand.* 1–26. 10.1520/D6682-08 (2010).

[32] Leturia M, Benali M, Lagarde S, Ronga I, Saleh K (2014). Characterization of flow properties of cohesive powders: a comparative study of traditional and new testing methods. Powder Technol..

[33] Strondl A, Lyckfeldt O, Brodin H, Ackelid U (2015). Characterization and control of powder properties for additive manufacturing. JOM.

[34] Søgaard SV, Pedersen T, Allesø M, Garnaes J, Rantanen J (2014). Evaluation of ring shear testing as a characterization method for powder flow in small-scale powder processing equipment. Int. J. Pharm..

[35] Clayton J, Millington-Smith D, Armstrong B (2015). The application of powder rheology in additive manufacturing. JOM.

[36] Schulze D (2011). Round robin test on ring shear testers. Adv. Powder Technol..

[37] Shi H (2018). Effect of particle size and cohesion on powder yielding and flow. KONA Powder Part. J..

[38] Koynov S, Glasser B, Muzzio F (2015). Comparison of three rotational shear cell testers: powder flowability and bulk density. Powder Technol..

[39] Salehi H, Barletta D, Poletto M (2017). A comparison between powder flow property testers. Particuology.

[40] Gelnar, D., Zegzulka, J., Soos, L. & J. D. Validation device and method of static and dynamic angle of repose measurement. (2013).

[41] Samantha SC (2015). Drying by spray drying in the food industry: Micro-encapsulation, process parameters and main carriers used. Afr. J. Food Sci..

[42] USP. U. S. Pharmacopoeia National Formulary. In *United States Pharmacopeial, 2011* (2012).

[43] Jezerská, L., Zádrapa, F., Žurovec, D. & Zegzulka, J. Avalanching and aeration regions for glidants. In *NANOCON 2017—Conference Proceedings, 9th International Conference on Nanomaterials—Research and Application* vols 2017-October 781–786 (2018).

[44] Tan JH, Wong WLE, Dalgarno KW (2017). An overview of powder granulometry on feedstock and part performance in the selective laser melting process. Addit. Manuf..

[45] Zegzulka J (2018). Internal friction angle of metal powders. Metals (Basel).

[46] Jenike A, Johanson J (1975). Storage and flow of solids. Powder Technol..

[47] Mihlbachler K, Kollmann T, Seidel-Morgenstern A, Tomas J, Guiochon G (1998). Measurement of the degree of internal friction of two native silica packing materials. J. Chromatogr. A.

[48] Brookfield Engineering Laboratories Inc. *Brookfield Powder Flow Tester: Operating Instructions,* vol. 8139 (2014).

[49] Berry RJ, Bradley MSA, McGregor RG (2015). Brookfield powder flow tester—results of round robin tests with CRM-116 limestone powder. Proc. Inst. Mech. Eng. Part E J. Process Mech. Eng..

[50] Freeman R (2007). Measuring the flow properties of consolidated, conditioned and aerated powders—a comparative study using a powder rheometer and a rotational shear cell. Powder Technol..

[51] Engeli R, Etter T, Hövel S, Wegener K (2016). Processability of different IN738LC powder batches by selective laser melting. J. Mater. Process. Technol..

[52] Geldart D, Abdullah EC, Hassanpour A, Nwoke LC, Wouters I (2006). Characterization of powder flowability using measurement of angle of repose. China Particuol..

[53] Farley R, Valentin FHH (1968). Effect of particle size upon the strength of powders. Powder Technol..

[54] Krantz M, Zhang H, Zhu J (2009). Characterization of powder flow: static and dynamic testing. Powder Technol..

[55] Macho O (2020). Analysis of static angle of repose with respect to powder material properties. Acta Polytech..

